# Total plasma homocysteine, folate, and vitamin b12 status in healthy Iranian adults: the Tehran homocysteine survey (2003–2004)/a cross – sectional population based study

**DOI:** 10.1186/1471-2458-6-29

**Published:** 2006-02-13

**Authors:** Hossein Fakhrzadeh, Sara Ghotbi, Rasoul Pourebrahim, Masoumeh Nouri, Ramin Heshmat, Fatemeh Bandarian, Alireza Shafaee, Bagher Larijani

**Affiliations:** 1 Assistant Professor of Cardiology, Endocrine and Metabolism Research Center, Tehran University of Medical Sciences, Tehran, Iran; 2 Researcher, Endocrine and Metabolism Research Center, Tehran University of Medical Sciences, Tehran, Iran; 3 PhD candidate of Epidemiology, Epidemiology and Biostatistics Dep., Public Health School, Tehran University of Medical Sciences, Tehran, Iran; 4 PhD candidate of Health Services Management, Endocrine and Metabolism Research Center, Tehran University of Medical Sciences, Tehran, Iran; 5 MD laboratory, Researcher, Endocrine and Metabolism Research Center, Tehran University of Medical Sciences, Tehran, Iran; 6 Professor of Internal Medicine and Endocrinology, Endocrine and Metabolism Research Center, Tehran University of Medical Sciences, Tehran, Iran

## Abstract

**Background:**

Elevated plasma total homocysteine is an independent risk factor for cardiovascular disease and a sensitive marker of the inadequate vitamin B12 and folate insufficiency. Folate and vitamin B12 have a protective effect on cardiovascular disease. This population based study was conducted to evaluate the plasma total homocysteine, folate, and vitamin B12 in healthy Iranian individuals.

**Methods:**

This study was a part of the Cardiovascular Risk Factors Survey in the Population Lab Region of Tehran University has been designed and conducted based on the methodology of MONICA/WHO Project. A total of 1214 people aged 25–64 years, were recruited and assessed regarding demographic characteristics, homocysteine, folate, and vitamin B12 levels with interview, questionnaires, examination and blood sampling. Blood samples were gathered and analyzed according to standard methods.

**Results:**

The variables were assessed in 1214 participants including 428 men (35.3%) and 786 women (64.7%). Age-adjusted prevalence of hyperhomocysteinemia (Hcy≥15 μmol/L) was 73.1% in men and 41.07% in women (P < 0.0001). Geometric mean of plasma homocysteine was 19.02 ± 1.46 μmol/l in men and 14.05 ± 1.45 μmol/l in women (P < 0.004) which increased by ageing. Age-adjusted prevalence of low serum folate level was 98.67% in men and 97.92% in women.  Age-adjusted prevalence  of  low  serum  vitamin  B12  level  was  26.32%  in men  and 27.2% in women. Correlation coefficients (Pearson's r) between log tHcy and serum folate, and vitamin B12 indicated an inverse correlation (r = -0.27, r = -0.19, P < 0.0001, respectively).

**Conclusion:**

These results revealed that the prevalence of hyperhomocysteinemia, low folate and vitamin B12 levels are considerably higher than other communities. Implementation of preventive interventions such as food fortification with folic acid is necessary.

## Background

Homocysteine (Hcy) is a nonessential sulfur-containing amino acid formed from the demethylation of an essential amino acid, methionine [1]. Plasma folate and vitamin B12 influence homocysteine metabolism as cosubstrate and cofactor, respectively [2]. Elevated plasma total homocysteine (tHcy) has been linked both to the inadequate status of vitamin cofactors (i.e. folate, vitamin B12 and B6) and to genetic defects in enzymes involved in homocysteine metabolism [3]. Genetic causes are mostly defects in the enzymes that control homocysteine metabolism. It is now believed that metabolism of homocysteine may be race and ethnic dependent [4].

Elevated plasma levels of homocysteine increase the risk for atherosclerosis, stroke, myocardial infarction, possibly Alzheimer's disease, cognitive impairment in the elderly, birth defects in pregnant women, and all-cause mortality [5]. Besides, hyperhomocysteinemia (HHcy) may induce changes in DNA that may result in procarcinogenic effects [6]. On one hand, plasma homocysteine is a very sensitive marker of folate and vitamin B12 status; plasma homocysteine levels are inversely related to plasma levels of these substances. The increase in homocysteine level occurs long before classic deficiency of folate and vitamin B12 become evident [7]. Inadequate levels of these vitamins have important health consequences that may be independent of their role in homocysteine metabolism [8]. Folate and vitamin B12 status has been related to the occurrence of neural tube defects. Other potential manifestations of folate deficiency include neurological and neuropsychiatric disorders, and preneoplastic conditions. Furthermore, folate deficiency has been associated with a predisposition to atherosclerotic cardiovascular disease [9,10]. Folate status may be negatively influenced by inadequate intake, genetic polymorphisms and interactions with various drugs [6]. Folate and vitamin B12 have a protective association with cardiovascular disease that can be partly explained by mechanisms independent of homocysteine, as suggested by several recent studies [11,12]. On the other hand, folate and cobalamin status are important modifiable determinants of plasma total homosysteine in the general population, and negative relations between plasma total homocysteine concentrations and these vitamins are observed even within their established normal and subnormal concentration ranges [13]. Testing for hyperhomocysteinemia may therefore be useful to assess the nutritional status in humans. Estimation of the proportion of cases with high homocysteine concentrations that can be attributed to inadequate vitamin status is complicated by the lack of a standard definition of a high total homocysteine concentration. In the absence of a definition based on increased risk for an adverse health outcome, such as vascular disease, upper reference limits from samples of healthy persons without established risk factors for high homocysteine concentrations have been used to define a high total homocysteine concentration [13-16].

Although the distribution of plasma concentrations of homocysteine has been reported in some populations, there is little available information describing homocysteine concentrations in the healthy Iranians [17]. In the present study, total homocysteine, folate, and vitamin B12 concentrations were measured in 1214 healthy Iranians as part of "Tehran Homocysteine Survey". This study describes the distributions of total homocysteine, folate, and vitamin B12 concentrations altogether in a sample of healthy Iranians.

## Methods

### Study design

The present data are part of a cross – sectional study (designed as Cardiovascular Risk Factor Survey in the Population Lab Region of Tehran University) arranged and conducted based on methodology of MONICA (Multinational Monitoring of Trends and Determinants in Cardiovascular Diseases)/WHO (World Health Organization) project [1,19] by EMRC (Endocrinology and Metabolism Research Center) affiliated with Tehran University of Medical Sciences (TUMS). We recruited 1573 apparently healthy residents of 17th district of Tehran – capital city of Iran – randomly chosen by one stage cluster random sampling from a group of 255337 people. This south western district of Tehran is the most crowded one, with low income inhabitants. Because of its unique characteristics, there has been selected as Tehran University Population Lab Region.

### Subjects and study criteria

Totally, 1573 healthy (without any history of severe renal, liver and cardiac dysfunction) participants (615 men and 958 women) aged 25–64 years were recruited: 140 were not interested to take part in this part of study, 152 did not meet the inclusion criteria (being healthy and 25–64 years old), and 67 had incomplete laboratory data.

Therefore, our analyses are based on 1214 participants (428 men and 786 women) with complete data on plasma total homocysteine, folate, and vitamin B12 concentrations. Exclusion criteria included known coronary heart disease (CHD), systemic illness, serious organ disease, proliferative and endocrine diseases, alcoholism, current pregnancy, current use of vitamins or other supplements, anticonvulsant and anticancer therapy. Participants underwent a standardized medical history, physical examination, anthropometric measurements and laboratory tests. Personal and lifestyle information were obtained by using modified MONICA questionnaires [18]. All respondents gave their written informed consent.

Our research protocol was approved by the EMRC ethics committee and was conformed to the principles embodied in the declaration of Helsinki.

### Laboratory analyses

Fasting blood samples were obtained in the early morning hours after 12 hours fast. The plasma samples were put in a cooled container and immediately carried to the EMRC laboratory, where the plasma was separated within 2 hours of sampling by centrifugation (20 min, RT, at 2000 RPM) and aliquots were stored at -70°c until determinations. In this part of the study, the following parameters were analyzed: total plasma homocysteine, serum vitamin B12 and serum folate. Plasma total homocysteine concentrations (the sum of homocysteine and homocysteine-cysteine mixed disulfides, free and protein-bound) were determined on frozen samples by HPLC (High-performance Liquid Chromatography) method (KNAUER, Germany), coupled with fluorescence detector. The method has been validated over a linearity range of 1–100 μmol/L from plasma. The intra-assay and inter-assay coefficient of variation for homocysteine samples were 3.9% and 5.8% respectively. Folate and vitamin B12 were measured simultaneously in the frozen serum aliquot by a double labeled radioassay kit (ICN Pharmaceuticals, New York). The intra-assay coefficient of variation for folate and vitamin B12 were 6.9% and 6.1%, respectively. The inter-assay coefficient of variation for folate and vitamin B12 were 7.5% and 6.8%, respectively.

### Statistical analyses

The distributions of total homocysteine, folate and vitamin B12 concentrations were skewed to the right. Therefore values were logarithmically natural transformed (Ln) to promote normality and presented as geometric mean and 95% confidence intervals. Various quartiles of homocysteine were estimated in analyses stratified according to the strongest predictors of mean total homocysteine concentrations in multivariate analysis: sex, age, folate score and vitamin B12 score. All values were calculated as mean ± SD (standard deviation) for the total group and separately by sex and age. Associations between categorical variables were reported by Chi-square test. Continuous variables were compared by Student's t test. A *P *value < 0.05 was considered significant. Data were analyzed by using Statistical Package for Social Sciences (SPSS) version 11.5 software.

A folate concentration less than 11 nmol/L [8,20,21] and a vitamin B12 concentration less than 185 pmol/L[8,22] served as the criterion for defining low vitamin levels. We also defined hyperhomocysteinemia (HHcy) as Table 1 demonstrates.

## Results

The group studied contained 428 (35.3%) male and 786 (64.7%) female 25–64-y-old individuals. Table 2 shows the clinical characteristics, nutritional state and other risk factors in the study participants.

Proportion of individual in each homocysteine classes has been shown in table 3. Hyperhomocysteinemia (tHcy≥ 15 μmol/L) was detected in 313 (crude prevalence: 73.1%, age-adjusted: 73.1%) males and 330 (crude prevalence: 42%, age-adjusted: 41.07%) females which was significantly higher in men than women (*P *< 0.0001). Although crude and age-adjusted prevalence of low folote levels (<11 nmol/L) were observed respectively in 98.8% and 98.67% of males and 97.9% and 97.92% of females, the difference between sexes was not statistically significant (*P *= 0.281). Crude and age-adjusted prevalence of low vitamin B12 levels (<185 pmol/L) were determined respectively in 26.8% and 26.32% of male and 27.3% and 27.2% of female individuals but the difference was not statistically significant either (*P *= 0.846).

Crude prevalence of Hyperhomocysteinemia for 45–54-year and 55–64-year-old age groups among both males and females were 79.1%, 87.4% and 44.6%, 57.1%, respectively (Table 4).

Notably, 12% (n = 146) of participants had normal total homocysteine concentrations, but mild and moderate hyperhomocysteinemia were detected in 47.6% (n = 578) and 5.4% (n = 65) of individuals, respectively. Crude, age and sex-adjusted prevalence of hyperhomocysteinemia in total participants were 53% and 56.2%, respectively. Crude prevalence of low folate, and vitamin B12 levels were detected in 98.2% (age and sex adjusted: 98.3%) and 27.1% (age and sex-adjusted: 26.77%) of total subjects respectively.

Correlation coefficients (Pearson's r) between log tHcy and serum folate, and vitamin B12 indicated an inverse correlation (*r *= -0.27, *r *= -0.19, *P *< 0.0001, respectively).

The geometric mean of total homocysteine level is the highest in 55–64-year-old age group as Table 5 presents. But in this age group, men had higher homocysteine concentrations than women.

The lowest level of serum folate was considered in both male and female 45–54-year age groups. The lowest level of vitamin B12 was determined in male 45–54-year-old and female 35–44-year-old age groups. In general, geometric mean of homocysteine was higher in men than women, and increased by age, but geometric mean of serum folate in all age groups were higher in men than women. Geometric mean of serum vitamin B12 was higher in all female age groups (except 35–44-year-old) than similar male age groups. Overall geometric mean of total homocysteine, folate, and vitamin B12 levels were 15.64 ± 1.50 μmol/L, 3.94 ± 1.67 nmol/L, 262.88 ± 1.82 pmol/L, respectively.

Men had significantly higher mean plasma homocysteine concentration than women (geometric mean = 19.02 ± 1.46 μmol/L, compared with 14.05 ± 1.45 μmol/L, *P *= 0.004) and lower mean serum folate levels than women (geometric mean = 3.66 ± 1.65 nmol/L compared with 4.1 ± 1.67 nmol/L, *P *< 0.0001). Mean serum vitamin B12 was not significantly higher in women (geometric mean = 269.27 ± 1.75 pmol/L) than in men (geometric mean = 251.39 ± 1.94 pmol/L, *P *= 0.063).

Distribution of participants according to their total homocysteine, folate, and vitamin B12 level quartiles (Q1–Q4) are displayed in Table 6.

By increasing age, there was a decreasing frequency in first quartile of homocysteine, while in fourth of homocysteine quartile in both sexes, an increasing frequency was considered by aging.

Regarding homocysteine quartiles, the highest distribution was found in male 55–65-year-old and female 25–34-year-old age groups. According to folate quartiles, the highest distribution was seen in male 45–54-year-old and female 55–64-year-old age groups.

Thresholds of each tHcy, folate and vitamin B12 quartiles were: 12.20, 2.80 and 174.00 μmol/L; 15.00, 4.00 and 285.00 μmol/L; 20.20, 5.40 and 378.00 μmol/L, respectively.

## Discussion

Many studies have shown that elevated total homocysteine concentration is an independent risk factor for cardiovascular diseases [24-27]. Total plasma homocysteine levels are higher in men than in women and at older ages [25,23]. Folate and vitamin B12 are the major nutritional determinants of homocysteine levels [28]. Persons with low circulating folate or vitamin B12 concentrations have higher fasting total homocysteine concentrations [13,23]. Elevated fasting homocysteine concentrations in turn, are usually normalized by treatment with folic acid and vitamin B12 [29,30]. Although epidemiologic surveys have determined total homocysteine concentrations in order to identify the prevalence of hyperhomocysteinemia, estimation of these cases is complicated by the lack of a standard definition of a high total homocysteine concentration [8,15,16,30].

In our study, the prevalence of hyperhomocysteinemia and low folate and vitamin B12 levels were higher than other populations [2,8,16,17,31]. Prevalence of hyperhomocysteinemia and the mean homocysteine concentrations were higher in men than in women and at older ages. The age and sex differences in total plasma homocysteine levels observed in this study are consistent with observations from published studies in large and small sample size of adult men and women in some reported populations [32,33]. Plasma homocysteine concentrations are increased with age and higher in men than in women [25,31,34,35].

For every 20 years of age, Hcy increases on average by 1.3 μmol/L [36]. The reasons for higher homocysteine concentrations at older ages are not well understood, although changes in renal function and impaired renal metabolism of homocysteine may be involved [37,38]. Furthermore, elevated plasma homocysteine concentrations in older populations can also be attributed to low blood folate concentrations [39] and an increased incidence of vitamin B12 deficiency resulting from malabsorption of vitamin B12 by the aging gut [40]. Men have average 1 μmol/L higher Hcy values than women [34,36]. Higher homocysteine concentrations in males than in females could be due to larger muscle mass and greater creatine phosphate synthesis in men, [1] lowering effect of estrogens in women [41] and differences in vitamin status [25] and homocysteine formation between sexes [1]. Part of the relationship with age in women might be explained by menopause, since the homocysteine concentration was found to be higher in post-menopausal women compared with premenopausal women [34].

In present study, mean total homocysteine concentration is notably higher than what other surveys have reported [2,8,16,17,31]. In addition, we observed that the prevalence of mild hyperhomocysteinemia (15< Hcy <30 μmol/L) was 47.6%. Probably, the condition of homocysteine distribution and concentration of values around ranges 10–30 μmol/L and low prevalence of normal total homocysteine concentrations in this sample, caused increase in the mean homocysteine level. In other words, although the mean homocysteine level is high, the severity of hyperhomocysteinemia is not that much. Furthermore, serum folates, and vitamin B12 levels compared to values from various countries, indicate that prevalence of low levels of folate, and vitamin B12 is higher in our sample [2,8,16,17,31]. It could be due to geographical variations, racial and ethnic differences [17], genetic causes, different lifestyle, and inadequate intake of B vitamins and folate, inaccurate cooking of vegetables and not implementing fortification of grain products with folic acid in our country. As it has been shown in table 1 it seems that low daily intake of vitamin B12 and folic acid is the eventual cause of low folate levels in our study population. Prolonged cooking of vegetables may destroy up to 90% of folate content [42]. Candidate genes can regulate plasma homocysteine concentrations, especially methylene tetrahydrofolate reductase (MTHFR) and cystathionine-β-synthase (CBS) genes. Recent studies have shown the importance of DNA polymorphisms in the genes for enzymes involved in homocysteine metabolism [31]. Moreover, it has been reported that rural-to-urban migrant population living in urban slums undergoing stressful socio-economic transition are likely to have low intakes of folate, and vitamin B12, which may have an adverse impact on plasma levels of homocysteine [43].

Limitations of the present investigation include not assessed several factors that might be associated to elevated tHcy, such as creatinine and vitamin B6 concentrations.

Our results confirm the findings of other observations that elevated total homocysteine concentrations are potentially attributable to low vitamin levels. Because folate is an established predictor of homocysteine concentration, it would be useful to investigate the effect of folate enrichment of foods on homocysteine concentration in the current Iranian population. Although firm therapeutic guidelines are lacking, consideration should be given to treatment as a primary prevention measure for patients whose baseline homocysteine level exceeds 15 μmol/L. Folic acid supplementation in a dose of 400–800 mg/day effectively lowers plasma homocysteine levels in a significant percentage of individuals, with the higher dose being needed by many to achieve a meaningful reduction in plasma homocysteine. However some patients will not respond to this dose of folate. This makes close follow-up and periodic monitoring of homocysteine levels essential. The only way to truly know if folate treatment is being effective is to recheck the homocysteine level [44]. This guideline could be followed by family physicians. Another essential prevention guideline is planning for fortification of enriched grain products with folic acid in Iran like many other countries [45]. The main motivation behind fortification is to abate the occurrence of neural tube defects (NTDs), a birth defect shown to be responsive to folic acid administration [46]. A secondary benefit of fortification might be a reduction in the incidence of cardiovascular disease [47] and probably certain cancers, [48] the occurrence of which are associated with low folate status. The success of fortification was quickly seen [45] with the declining incidence of folate deficiency and a concurrent decrease in the incidence of elevated plasma total homocysteine [49].

Although the distribution of plasma concentrations of homocysteine, folate, and vitamin B12 has been reported in some populations of other countries, there is little or no available information describing these parameters in healthy Iranians and their relations to gender and age. There is only one study that has reported homocysteine levels in Iranians. In this study, 402 participants 15 years of age or older of southwest of Iran were monitored and the mean plasma homocysteine level was reported significantly higher in men than women [17].

The cost effectiveness of assessment of homocysteine as a cardiovascular risk factor is important. The cost for homocysteine measurement is higher than other risk factors at individual level. However, since it has been reported as a highly potential risk factor of CHD [1,4,11] which can be modified easily by food supplementation with folate and vitamin B12, its assessment is valuable and has cost benefit at the public health level.

In conclusion, we found the association between plasma homocysteine concentration and age and sex, and blood levels of vitamins by using data from a cross-sectional study based on MONICA/WHO similar to other studies worldwide. Also, we reported that plasma homocysteine concentration was inversely associated with folate and vitamin B12 levels. The variables sex and age were not independently associated with homocysteine levels when other variables were considered. Because this study used cross-sectional data, it was not possible to evaluate the data in terms of cause-and-effect relations.

## Conclusion

So far, little has been published on distribution of total homocysteine concentrations in healthy Iranians. This study describes the distributions of total homocysteine, folate, and vitamin B12 concentrations altogether in a sample of healthy Iranians. According to significance of genetic and ethnic factors on total homocysteine level, we are conducting a study to observe Iranians' MTHFR (methylene tetrahydrofolate reductase) and CBS (cystathionine-β-synthase) polymorphisms. In addition, our clinical trials are underway to investigate the impact of folic acid supplements on plasma homocysteine concentrations and hypertension in Iranian population. Since there are little or no available information in our country, it is possible that future studies could reveal excess determinants of hyperhomocysteinemia.

## List of abbreviations

**CBS**: cystathionine-β-synthase, **CHD**: coronary heart disease, **EMRC**: Endocrinology and Metabolism Research Center, **Hcy**: homocysteine, **HHcy**: hyperhomocysteinemia, **HPLC**: High-performance Liquid Chromatography, **MONICA**: Multinational Monitoring of Trends and Determinants in Cardiovascular Diseases, **MTHFR**: methylene tetrahydrofolate reductase, **NTDs**: neural tube defects, **tHcy**: plasma total homocysteine, **RPM**: round per minute, **RT**: room temperature, **TUMS**: Tehran University of Medical Sciences, **WHO**: World Health Organization.

## Competing interests

The author(s) declare that they have no competing interests.

## Authors' contributions

1) HF: principle investigator, has made substantial contributions to conception and design of the study

2) SG; has made substantial contributions to data acquisition and has been involved in drafting and interpretation of data

3) RP: co-principle investigator has made substantial contributions to data acquisition

4) MN has made substantial contributions to data acquisition

5) RH has made substantial contributions to analysis, interpretation and revising of data

6) AS; has made substantial contributions to data acquisition

7) FB: critical revision, data analysis and substantial contribution

8) BL; has given final approval of the version to be published

## Pre-publication history

The pre-publication history for this paper can be accessed here:



## Figures and Tables

**Table 1:** Based Homocysteine Ranges in Our Study [23]

**Normal**	tHcy < 15 μmol/L
**Mild HHcy**	tHcy 15 – 30 μmol/L
**Moderate HHcy**	tHcy 30 – 100 μmol/L
**Severe HHcy**	tHcy > 100 μmol/L

**Table 2:** Clinical characteristics, risk factors and nutritional state of the study population

**Characteristic**	**Male**	**Female**	**P-value**
**Age**	41.9 ± 12.6	40.8 ± 11.7	0.15
**BMI (kg/m^2^)**	26.5 ± 7.3	29.2 ± 9.2	0.0001
**Cigarette Smoking**	31.7%	5.7%	0.0001
**Diabetes**	9.7%	11.8%	0.23
**Total cholesterol (mg/dl)**	187 ± 39.5	198 ± 46.1	0.0001
**LDL(mg/dl)**	96.5 ± 26.2	102.4 ± 30	0.004
**HDL(mg/dl)**	54.2 ± 16.3	61.1 ± 18	0.0001
**Triglyceride(mg/dl)**	196.8 ± 174.4	184.3 ± 141.4	0.1
**HTN**	46.4%	39.2%	0.01
**Vitamin B12 intake (μgr/day)**	2.9 ± 2.6	2.2 ± 2.07	0.01
**Folic acid intake (μgr/day)**	294 ± 73.7	270 ± 60.7	0.05

**Table 3:** Proportion of individual in each homocysteine classes according sex and age groups

	**Age range**	**<10**	**10–15**	**15–30**	**30–100**	**>100**
**Male**	25–34	5.5%	28.5%	55.2%	10.9%	0
	35–44	5%	27.7%	60.4%	6.9%	0
	45–54	0%	20.9%	67.2%	11.9%	0
	55–64	1.1%	11.6%	73.7%	13.7%	0
**Female**	25–34	21.5%	45%	31.1%	2.3%	0
	35–44	15.7%	41.65	40.5%	2.2%	0
	45–54	17.5%	38%	41.6%	3%	0
	55–64	10.5%	32.3%	54.9%	2.3%	0

**Table 4:** Prevalence of Hyperhomocysteinemia and Low Vitamin Concentrations According to Sex and Age Group

**sex**	**Age-Group(Y)**	**N**	**HHcy (%)**	**Low Folate (%)**	**Low Vitamin B12 (%)**
**Male**	25–34	165	66.1% (109)	98.7	24.5
	35–44	101	67.3% (68)	97.8	26.1
	45–54	67	79.1% (53)	100.0	27.4
	55–64	95	87.4%(83)	98.9	31.1
**Female**	25–34	302	33.4% (101)	97.9	24.7
	35–44	185	42.7% (79)	98.3	28.7
	45–54	166	44.6% (74)	98.1	31.2
	55–64	133	57.1% (76)	96.9	26.6

**Table 5:** Mean Total Homocysteine, Folate and Vitamin B12 According to Sex and Age Group

**Characteristics**	**Male Age Group (Y)**				**Female Age Group (Y)**			
	**25–34**	**35–44**	**45–54**	**55–64**	**25–34**	**35–44**	**45–54**	**55–64**
**Participants (N)**	165	101	67	95	302	185	166	133

**Geometric mean** **Hcy μmol/L** **CI 95%**	18.0 16.9–19.1	18.5 17.2–19.9	19.5 17.9–21.1	21.3 19.9–22.8	13.3 12.8–13.9	14.1 13.3–14.9	14.2 13.4–15	15.6 14.7–16.7

**Geometric mean** **Folate nmol/L** **CI 95%**	3.6 3.4–3.9	3.7 3.3–4.2	3.3 2.9–3.7	3.9 3.6–4.3	4.0 3.8–4.3	4.1 3.8–4.5	4.0 3.7–4.3	4.3 4.0–4.7

**Geometric mean** **Vit B12 nmol/L** **CI 95%**	255.8 232–281	262.6 224–308	235.1 194–284	244.1 216–276	256.1 259–294	276.5 236–278	264.8 241–290	278.0 251–307

**Table 6:** Distribution of Participants in Each Sex and Age Group Based on Their tHcy, Folate and Vitamin B12 Quartiles

		**tHcy Quartiles(%)**				**Folate Quartiles (%)**				**Vitamin B12 Quartiles (%)**			
**Sex**	**Age Group (y)**	**1**	**2**	**3**	**4**	**1**	**2**	**3**	**4**	**1**	**2**	**3**	**4**

**Male**	25–34	17.7	19.5	25.6	37.2	29.0	27.1	25.2	18.7	23.3	23.3	32.1	21.4
	35–44	11.9	20.8	23.8	43.6	27.8	23.3	24.4	24.4	25.3	17.6	33.0	24.2
	45–54	1.5	26.9	32.8	38.8	40.0	25.0	23.3	11.7	25.8	27.4	25.8	21.0
	55–64	3.2	11.6	28.4	56.8	28.1	20.2	24.7	27.0	30.3	29.2	18.0	22.5
**Female**	25–34	36.9	32.9	18.3	12.0	22.3	26.6	23.8	27.3	22.6	21.6	28.9	26.8
	35–44	33.0	26.5	25.9	14.6	20.5	27.5	21.6	30.4	24.9	33.3	15.8	26.0
	45–54	32.9	26.1	24.8	16.1	23.2	22.6	23.9	30.3	28.4	20.6	25.2	25.8
	55–64	19.5	26.3	31.6	22.6	18.2	21.5	26.4	33.9	23.0	23.0	25.4	28.6
